# Clinical genomic profiling to identify actionable alterations for investigational therapies in patients with diverse sarcomas

**DOI:** 10.18632/oncotarget.16845

**Published:** 2017-04-05

**Authors:** Roman Groisberg, David S. Hong, Vijaykumar Holla, Filip Janku, Sarina Piha-Paul, Vinod Ravi, Robert Benjamin, Shreyas Kumar Patel, Neeta Somaiah, Anthony Conley, Siraj M. Ali, Alexa B. Schrock, Jeffrey S. Ross, Philip J. Stephens, Vincent A. Miller, Shiraj Sen, Cynthia Herzog, Funda Meric-Bernstam, Vivek Subbiah

**Affiliations:** ^1^ Department of Investigational Cancer Therapeutics (A Phase I Program), Division of Cancer Medicine, The University of Texas MD Anderson Cancer Center, Houston, Texas 77030, USA; ^2^ Division of Cancer Medicine, The University of Texas MD Anderson Cancer Center, Houston, Texas 77030, USA; ^3^ Khalifa Institute for Personalized Cancer Therapy (IPCT), The University of Texas MD Anderson Cancer Center, Houston, Texas 77030, USA; ^4^ Department of Sarcoma Medical Oncology, Division of Cancer Medicine, The University of Texas MD Anderson Cancer Center, Houston, Texas 77030, USA; ^5^ Division of Pediatrics, The University of Texas MD Anderson Cancer Center, Houston, Texas 77030, USA; ^6^ Foundation Medicine Inc, Cambridge, Massachusetts 02139, USA

**Keywords:** sarcoma, targeted therapy, phase I trials

## Abstract

**Background:**

There are currently no United States Food and Drug Administration approved molecularly matched therapies for sarcomas except gastrointestinal stromal tumors. Complicating this is the extreme diversity, heterogeneity, and rarity of these neoplasms. Few therapeutic options exist for relapsed and refractory sarcomas. In clinical practice many oncologists refer patients for genomic profiling hoping for guidance on treatment options after standard therapy. However, a systematic analysis of actionable mutations has yet to be completed. We analyzed genomic profiling results in patients referred to MD Anderson Cancer Center with advanced sarcomas to elucidate the frequency of potentially actionable genomic alterations in this population.

**Methods:**

We reviewed charts of patients with advanced sarcoma who were referred to investigational cancer therapeutics department and had CLIA certified comprehensive genomic profiling (CGP) of 236 or 315 cancer genes in at least 50ng of DNA. Actionable alterations were defined as those identifying anti-cancer drugs on the market, in registered clinical trials, or in the Drug-Gene Interaction Database.

**Results:**

Among the 102 patients analyzed median age was 45.5 years (range 8-76), M: F ratio 48:54. The most common subtypes seen in our study were leiomyosarcoma (18.6%), dedifferentiated liposarcoma (11%), osteosarcoma (11%), well-differentiated liposarcoma (7%), carcinosarcoma (6%), and rhabdomyosarcoma (6%). Ninety-five out of 102 patients (93%) had at least one genomic alteration identified with a mean of six mutations per patient. Of the 95 biopsy samples with identifiable genomic alterations, the most commonly affected genes were TP53 (31.4%), CDK4 (23.5%), MDM2 (21.6%), RB1 (18.6%), and CDKN2A/B (13.7%). Notable co-segregating amplifications included MDM2-CDK4 and FRS2-FGF. Sixteen percent of patients received targeted therapy based on CGP of which 50% had at least stable disease.

**Conclusions:**

Incorporating CGP into sarcoma management may allow for more precise diagnosis and sub-classification of this diverse and rare disease, as well as personalized matching of patients to targeted therapies such as those available in basket clinical trials.

## INTRODUCTION

Recurrent and metastatic sarcomas are a rare and heterogeneous group of diseases. With well over 70 subtypes, the exact diagnosis alone can be challenging to make [1]. When sarcomas progress beyond efficacious local control, the standard practice with few notable exceptions is to treat with cytotoxic agents until progression or intolerance [2]. Unfortunately, these cytotoxic agents yield overall response rates of around 25% [3, 4]. Over the last three decades recurrent translocations have been found that drive the development of certain sarcomas and are now used as an adjunctive diagnostic tool [5, 6]. Unfortunately, in clinical practice most sarcoma therapies are not yet targeting these unique and simple fusions. While the transcription factor fusions pose an enormous drug development challenge, the kinase fusions are potentially targetable with current technology. A notable exception is non-fusion genomic alterations in kinases, with gastrointestinal stromal tumors serving as the paradigm of druggable c-Kit alterations by imatinib [7, 8]. A separate sub-group of sarcomas have complex cytogenetic changes hallmarked by genomic instability and are not characterized by discrete gene fusions [9].

The promise of personalized medicine has become a realization for many malignancies. Pairing genomic alterations and targeted therapy has transformed diseases like lung cancer, leukemia, and breast cancer [10–12]. Outside of gastrointestinal stromal tumors, inflammatory myofibroblastic tumors, and PECOMAs [13], targeted therapies in sarcomas have not seen such breakthroughs. Perhaps it is the staggering heterogeneity of the disease, relative rarity, or difficulty making a definitive diagnosis outside tertiary care centers that makes it challenging [14–20]. To compound the problem, some of the sarcomas are a group of biologically complex and resistant diseases and, outside of surgically curable local disease, portend an exceptionally poor prognosis when metastatic [21, 22]. A large portion of these patients will be referred for clinical trials partly because of their young age, preserved performance status, or scarcity of treatment options [23, 24]. To date much has been published about potentially targetable alterations, but clinical translation in sarcoma has been minimal [25–31]. We undertook a systematic analysis of potentially druggable alterations in sarcomas based on comprehensive genomic profiling (CGP) performed in the course of clinical care and evaluated clinical response in patients receiving molecularly matched therapies. Here we present 102 sarcoma patients that were referred to the Investigational Therapeutics Department which is the phase 1 clinical trials program at MD Anderson Cancer Center.

## RESULTS

All patients in this study had advanced or metastatic relapsed or refractory sarcoma or did not have any other standard care therapies available when they presented for clinical trials. There were 48 men (47%) and 54 women (53%) in this patient cohort with a median age at diagnosis of 45.5 years (range 8-76). There were 36 patients (35%) who had primary site biopsied and sent for next generation sequencing (NGS) and 66 (65%) who had a metastatic site sent for NGS. Of the 102 patients, 38 were metastatic at time of diagnosis. The most common histologies seen in our study was leiomyosarcoma (8 uterine and 11 non-uterine, 18.6%), dedifferentiated liposarcoma (11%), osteosarcoma (11%), well-differentiated liposarcoma (7%), carcinosarcoma (6%), and rhabdomyosarcoma (6%) (Table 1). All tumors were reviewed and histology confirmed by MD Anderson pathology department. The most frequently seen genomic alterations are summarized in (Figure 1A). Ninety-five out of 102 patients (93%) had at least one genomic alteration identified with a mean of six alterations per patient. The vast majority (49%) of all alterations were amino acid substitutions. Amplifications were the second most common alteration (31%). The remaining alterations were split as described in (Figure 1B). The most commonly altered genes were TP53 (31.4%), CDK4 (23.5%), MDM2 (21.6%), RB1 (18.6%), and CDKN2A/B (13.7%) (Figure 1A).

**Table 1:** Patient characteristics

Patient Characteristics		
Median age at Dx	45.5	8-76 years
#Men	48	47.06%
#Women	54	52.94%
**Race**		
Caucasian	78	76.47%
AA	10	9.80%
Hispanic	13	12.75%
Asian	1	0.98%
**Histology**		
LEIOMYOSARCOMA	19	18.63%
DEDIFFERENTIATED LIPOSARCOMA	11	10.78%
OSTEOSARCOMA	11	10.78%
WELL DIFFERENTIATED LIPOSARCOMA	7	6.86%
CARCINOSARCOMA	6	5.88%
RHABDOMYOSARCOMA (NOS)	6	5.88%
GASTROINTESTINAL STROMAL TUMOR	5	4.90%
SPINDLE CELL SARCOMA	5	4.90%
SYNOVIAL SARCOMA	4	3.92%
ALVEOLAR SOFT PART SARCOMA	3	2.94%
CHONDROSARCOMA	4	3.92%
CHORDOMA	3	2.94%
CLEAR CELL SARCOMA	3	2.94%
EWING SARCOMA	3	2.94%
UNCLASSIFIED	3	2.94%
ALVEOLAR RHABDOMYOSARCOMA	2	1.96%
FIBROSARCOMA	2	1.96%
BRAIN GLIOSARCOMA	1	0.98%
DESMOPLASTIC SMALL ROUND CELL TUMOR	1	0.98%
PLEOMORPHIC SARCOMA	1	0.98%
MALIGNANT PERIPHERAL NERVE SHEATH	1	0.98%
MYXOIDLIPOSARCOMA	1	0.98%
	102	100.00%
Metastasis at diagnosis	38	
Metastasis at biopsy	86	
**Biopsy site**		
Primary	36	35%
Metastasis	66	65%

**Figure 1A:**
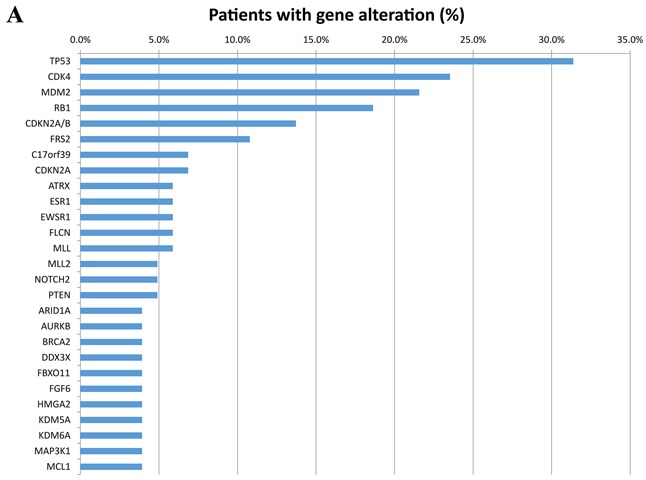
Frequency of the most common genes altered by percentage of 102 patients with diverse sarcomas Only alterations seen in at least 4% of patients are included. Different alteration in the same gene are listed under the same gene name.

**Figure 1B:**
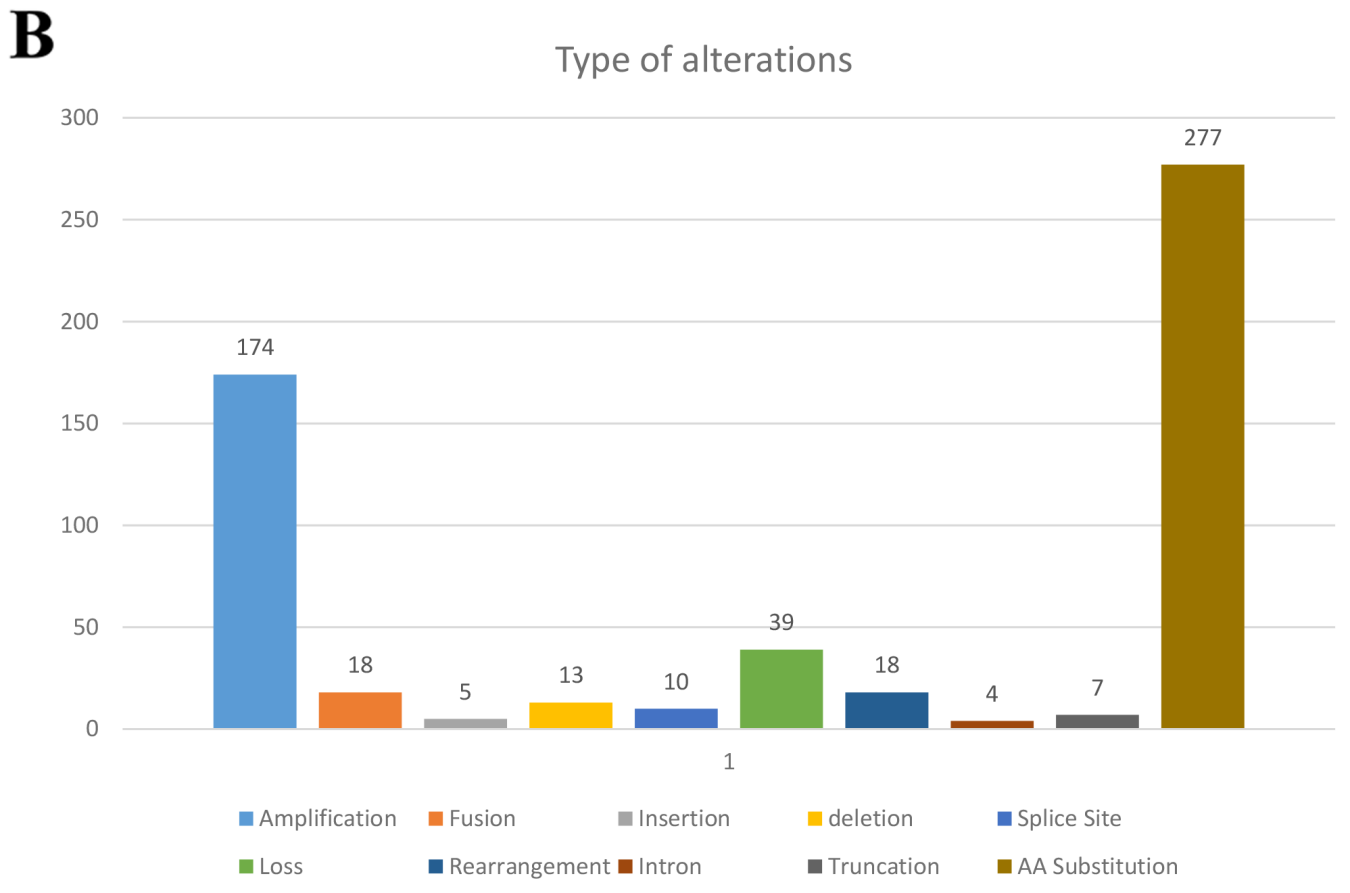
Types of gene alterations seen as a percentage of 102 patients with diverse sarcomas

Two of the most common alterations (MDM2 and CDK4 amplifications) are both actionable. MDM2 was altered in 22 patients and all were amplifications. All 22 of these patients also had CDK4 amplifications. One additional patient had a CDK4 amplification without MDM2 amplification. Ten of these co-amplified CDK4/MDM2 cases were dedifferentiated liposarcomas, seven were well-differentiated liposarcomas, two rhabdomyosarcomas (one pleomorphic and one nos), one osteosarcoma, one ewing sarcoma, and one unclassified soft tissue sarcoma. Four of the well-differentiated liposarcoma patients were treated with an investigational MDM2 inhibitor and all achieved at least stable disease, some showing a very durable response.

Other notable mutations include three FRS2 and FGF co-amplifications seen in a rhabdomyosarcoma, osteosarcoma, and dedifferentiated liposarcoma. The rhabdomyosarcomas were FOXO1 fusion-negative, hinting at a higher number of mutations [32]. We also identified previously reported fusions of SSX with SS18 in synovial sarcoma, as well as HMGA2 in liposarcoma. Three of the leiomyosarcomas had a mutation in the Lynch syndrome gene MSH2 (two uterine and one non-uterine). A complete list of all identified mutations can be seen in (Table 2).

**Table 2:** All identified mutations from the NGS panel

ABL1	CCT6B	FAM123B	IGF1R	MSH6	RANBP2	WHSC1
ACTB	CD274	FAM46C	IL7R	MTOR	RB1	WT1
AKT1	CD36	FANCA	INPP4B	MYC	RELN	YY1AP1
AKT2	CD70	FANCD2	INPP5D	MYCL1	RICTOR	ZNF703
AKT3	CDK12	FANCE	IRF2	MYO18A	ROS1	
ALK	CDK4	FAS	IRS2	MYST3	RUNX1	
APC	CDKN2A	FAT1	JAK1	NF1	RUNX1T1	
APH1A	CDKN2A/B	FBXO11	JAK2	NF2	SETD2	
AR	CEBPA	FBXW7	JAK3	NFKBIA	SMARCA1	
ARID1A	CHD2	FDF23	JUN	NKX2-1	SMARCA4	
ARID1B	CHEK2	FGF10	KDM5A	NOD1	SMARCB1	
ASXL1	CIC	FGF14	KDM5C	NOTCH1	SMC1A	
ATM	CIITA	FGF23	KDM6A	NOTCH2	SOCS2	
ATR	CPS1	FGF6	KDR	NRAS	SPOP	
ATRX	CREBBP	FGFR1	KEAP1	nsT	SPTA1	
AURKA	CSF1R	FGFR2	KIT	NTRK1	SSX	
AURKB	CTNNB1	FLCN	KRAS	NTRK3	SSX2	
BARD1	CUX1	FLT4	LRP1B	PAG1	STAG2	
BCL11B	DAXX	FLYWCH1	LYN	PAK3	STAT5B	
BCL2A1	DDIT3	FOXO3	MAFB	PALB2	STAT6	
BCL2L2	DDR2	FRS2	MALT1	PASK	STK11	
BCOR	DDX3X	gement	MAP2K2	PAX5	SUFU	
BCORL1	DNM2	GNA12	MAP2K4	PC	SYK	
BIRC3	DNMT3A	GNAS	MAP3K1	PCLO	TCL1A	
BLM	DOT1L	GPR124	MAP3K14	PDCD1LG2	TET2	
BRAF	DTX1	GRIN2A	MCL1	PDGFRA	TGFBR2	
BRCA1	EBF1	HDAC4	MDM2	PDGFRB	TLL2	
BRCA2	EGFR	HGF	MDM4	PIK3CA	TNFAIP3	
BRD4	EMSY	HIST1H1C	MED12	PIK3R1	TNFRSF17	
BTG1	EP300	HIST1H1D	MET	PIM1	TOP1	
C17orf39	EPHA5	HIST1H2AC	MIB1	PRDM1	TOP2A	
CARD11	EPHA7	HIST1H2AG	MKI67	PRKDC	TP53	
CBFB	EPHB1	HLGGSSCSTC	MLL	PTCH1	TSC1	
CBL	ERBB4	HMGA2	MLL2	PTEN	TSC2	
CCND1	ERG	HSP90AA1	MLL3	PTPN11	TSHR	
CCND2	ESR1	ICK	MPL	PTPRO	TYK2	
CCND3	EWSR1	IDH1	MSH2	RAD21	VHL	
CCNE1	EWSR1-NFATC2	IDH2	MSH3	RAD50	WDR90	

Of the 102 patients in our cohort, forty (39%) had either no reported mutation (7%) or no actionable mutation (32%). The remaining 62 (61%) patients all had a potentially actionable alteration. Fourteen (14%) patients had an alteration that could be targeted with an approved drug in sarcoma (on-label). This was either an off-target effect of pazopanib or imatinib and included five patients with PDGFR (1 GIST), four with FGFR, three with KIT (2 GIST), and two with KDR gene aberrations.

**Figure 2:**
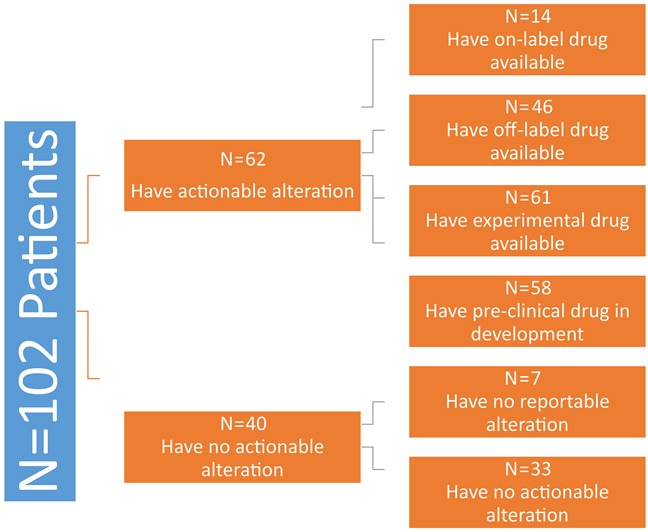
Number of sarcoma patients with actionable mutations divided by drug availability Patients had overlap between approved, off-label, and experimental drug options.

Forty-six (45%) patients had an alteration that could be targeted with a drug approved in another disease (off-label). Sixty-one (60%) patients had an alteration that could potentially be targeted by a drug currently available in clinical trials and, barring particular exclusion criteria, all of them could have been enrolled on a matching trial. Fifty-eight (57%) had an alteration for which a drug currently in pre-clinical development could be used (Figure 2).

**Figure 3:**
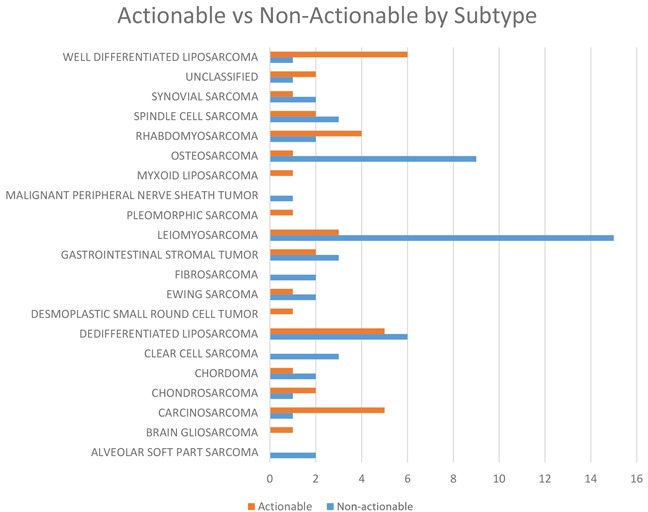
Number of patients with actionable as compared to non-actionable distributed by sarcoma subtype

A subtype analysis of all sarcoma types revealed that the probability of having an actionable mutation was related to histology (Figure 3). Notable sarcoma subtypes include dedifferentiated liposarcoma (100%), well-differentiated liposarcoma (100%), and carcinosarcoma (83%) all of which had an exceptional number of patients with actionable mutations. As noted above, dedifferentiated/well-differentiated liposarcomas had a preponderance of MDM2 and CDK4 mutations. Carcinosarcomas had targetable mutations in AKT2 and harbored a resistance mutation in ESR1 (Table 3).

**Table 3:** Actionable alteration by sarcoma subtype

Histology (patients)	No reportable alteration, n (%)	Patients had alteration(s), but none actionable, n (%)	Patients with approved drug(s) in the disease available, n (%) (on-label)	Patients with approved drug(s) in another disease available, n (%) (off-label)	Patients with experimental treatment options (clinical trials), n (%)	Patients with pre-clinical treatment options, n(%)
LEIOMYOSARCOMA	1	10	3	6	8	7
DEDIFFERENTIATED LIPOSARCOMA		0	0	11	10	10
OSTEOSARCOMA	1	6	1	2	4	4
WELL DIFFERENTIATED LIPOSARCOMA		0	1	7	7	7
CARCINOSARCOMA		0	3	5	6	6
RHABDOMYOSARCOMA		1	1	2	5	5
GASTROINTESTINAL STROMAL TUMOR		1	3	3	4	4
SPINDLE CELL SARCOMA		1	1	2	4	3
SYNOVIAL SARCOMA	1	2	0	1	1	1
ALVEOLAR SOFT PART SARCOMA	1	1	0	1	1	1
CHONDROSARCOMA	1	1	0	0	2	1
CHORDOMA		1	0	1	2	2
CLEAR CELL SARCOMA		3	0	0	0	0
EWING SARCOMA		2	0	1	1	1
UNCLASSIFIED		1	0	1	2	2
ALVEOLAR RHABDOMYOSARCOMA	2	0	0	0	0	0
FIBROSARCOMA		2	0	0	0	0
BRAIN GLIOSARCOMA		0	0	1	1	1
DESMOPLASTIC SMALL ROUND CELL TUMOR		0	0	1	1	1
PLEOMORPHIC SARCOMA		0	1	1	1	1
MALIGNANT PERIPHERAL NERVE SHEATH TUMOR		1	0	0	0	0
MYXOID LIPOSARCOMA		0	0	0	1	1
	7	33	14	46	61	58
	6.86%	32.35%	13.73%	45.10%	59.80%	56.86%

Clinical response was highly variable. Forty-three patients (42%) only received tumor sequencing, but did not participate in a clinical trial. The remaining fifty-nine patients (58%) chose to participate in a clinical trial. Of these, sixteen (16%) received therapy directed by molecular profile. Of these sixteen patients, eight (50%) had at least stable disease (Table 4).

**Table 4:** Results of sixteen sarcoma patients treated with targeted therapy based on NGS results

Patients treated with targeted therapy based on NGS result				
Histology	Gene	Mutation	Treatment and Best response	Comments and Referenes
BRAIN GLIOSARCOMA	BRAF	V600E	vemurafenib —> PR	86% decrease, duration of Response 16 months [44]
CARCINOSARCOMA	ESR1	A569T	anastrozole plus everolimus —> PD	IHC for PTEN was positive. ER was 3+ per IHC. ESR1 is a resistance mutation
DEDIFFERENTIATED LIPOSARCOMA	ROS1	amplification	ceritinib —> SD	Best response SD x5 months [20]
DEDIFFERENTIATED LIPOSARCOMA	MDM2	amplification	MDM2 inhibitor —> PR	Best response PR x3 cycles
GASTROINTESTINAL STROMAL TUMOR	KIT,AKT	amplification	Imatinib - PD sutent -PD, regorafenib-PD,AKT inhibitor —> PR	Best response PR, progressed after 22 cycles. Initially dx as wt kit and pdgfr, FM later showed akt, kit, mdm4, MCL1 amplification
LEIOMYOSARCOMA	ROS1	D1538V	pazopanib and crizotinib —> SD	SD x 6 months
LEIOMYOSARCOMA	PTEN	Loss	PI3K Inhibitor —> PD	Deceased after 3 days on study
LEIOMYOSARCOMA	ROS1	D1538V	pazopanib and crizotinib —> PD	Patient deceased prior to restaging scans
PLEOMORPHIC SARCOMA	ALK	MEMO1-ALK fusion	ceritinib —> PD	Progressed after 4 cycles [20]
MYXOID LIPOSARCOMA	AKT1	E17K	AKT inhibitor —> SD	Stopped after 1 cycle due to ggt elevation
OSTEOSARCOMA	PDGFRA	amplification	Sorafenib, Avastin, and Torisel —> PD	PD after 1 cycle [29]
SPINDLE CELL SARCOMA	BRAF	KIAA1549-BRAF fusion	Sorafenib, Avastin, and Torisel —> SD	Best response 28% reduction per RECIST. Also PTEN Loss. SD for 11 cycles, until death [31].
WELL DIFFERENTIATED LIPOSARCOMA	MDM2	amplification	MDM-2 —> SD	Best response SD x8 cycles
WELL DIFFERENTIATED LIPOSARCOMA	MDM2	amplification	MDM2 inhibitor —> CR	On since 2008, has had several resections during this period. Now NED again
WELL DIFFERENTIATED LIPOSARCOMA	MDM2	amplification	MDM2/MDMX inhibitor —> SD	Stopped after 2 cycles due to side effects
WELL DIFFERENTIATED LIPOSARCOMA	MDM2	amplification	MDM2 inhibitor —> SD	SD x23 months, stopped due to patient preference

All patients were treated on clinical trial. Eight patients had clinical benefit as defined by at least stable disease.

## DISCUSSION

Overall survival continues to be poor in metastatic sarcoma as a group. With small numbers and large diversity of subtypes, even the prospect of initiating and accruing a study in this population is daunting. Given the success of targeted therapy in other diseases, we sought to discover if CGP could aid in diagnosis and treatment of sarcomas as a whole. Using CGP we discovered that 61% of our patients had a potentially actionable mutation which could be targeted with either an off-label or an investigational therapeutic available in a clinical trial. A relative minority of samples had alterations targetable by on-label drugs (Pazopanib or imatinib). This is almost certainly driven by the paucity of approved (targeted) therapies in sarcoma. There was a skew toward certain histologic subtypes that harbor more potentially actionable mutations. Well-differentiated and dedifferentiated liposarcomas as well as carcinosarcomas stand out. Almost every patient who was a candidate for an off-label drug also had a drug available in a clinical trial. This speaks to the large variety of compounds available in trials, and the need to get sarcoma patients enrolled early and often.

One of the most frequent mutations seen in our cohort was MDM2 amplification. This was exclusively seen co-existing with a CDK4 amplification. This alteration has been reported most commonly in dedifferentiated/well-differentiated liposarcoma [33] and rarely in osteosarcoma [34]. In addition to the aforementioned subtypes our study detected this co-amplification in rhabdomyosarcoma, Ewing sarcoma (EWSR1 fusion positive), and an unclassified sarcoma. We hypothesize that this co-amplified duo may be more prevalent in other subtypes than previously thought. MDM2 and CDK4 have previously been proposed as tantalizing personalized targets in liposarcoma and indeed clinical trials are underway [35]. However, our small dataset suggests that such trials should be opened to all sarcoma subtypes and based on CGP rather than histology due to the occurrence of previously unreported mutations in the subtypes mentioned above.

Similar to the MDM2 and CDK4 co-amplification, we found FGF and FRS2 to be co-amplified. This has previously been observed in dedifferentiated liposarcoma and even found to be co-expressed with MDM2 and CDK4. This co-amplification is not surprising since FRS2 is the receptor substrate for FGF [36]. Despite this known overexpression in liposarcoma, to our knowledge this has never been reported in rhabdomyosarcoma or osteosarcoma. Given the relatively well-studied pathway, this FGF and FRS2 pathway is a salient potential target. Within the last few months, Ponatinib has been reported as a very potent inhibitor of this pathway in endometrial cancer [37]. Potentially, this serves as a novel therapeutic target in FRS2 and FGF co-amplified sarcomas.

While MDM2 and FRS2 are enticing for targeted therapy, our finding of MSH2 in leiomyosarcomas presents a potential for immunotherapy. MSH2 is an integral component of the mismatch repair machinery and causes microsatellite instability, creating a target for PD-1 blockade [38]. Successful treatment has been reported with PD-1 inhibitors in MSI-high colon cancer resulting in long-term disease control where chemotherapy had not been effective. MSH mutations have been reported in sarcomas previously, especially in uterine sarcomas [39]. While none of our three leiomyosarcoma patients received immunotherapy, this would have been a potentially useful therapy and opens up the possibility of a basket trial with all-comer MSI-high tumors treated with anti PD-1 drugs.

Previous studies have assessed genomic biomarker actionability [40, 41]. These studies included larger numbers of patients and reported high frequencies of clinically actionable genomic markers. However, we believe this is the first study to look specifically at sarcomas. We report significantly fewer actionable mutations (61%) than previous studies of other cancers (>90%) and this may be related to the fusion proteins-associated sarcomas which comprise approximately 30% of all sarcomas. Furthermore, this may suggest that many sarcomas are driven by copy number alterations rather than somatic mutations. It was encouraging to see that in our center genomic testing is being used to drive clinical decision in some patients. It was even more encouraging to see that almost half of those patients (47%) derived clinical benefit from mutational analysis based on at least stable disease as per RECIST.

MDM2 is a negative regulator of the tumor suppressor gene P53 and is a powerful oncogene. MDM2 and CDK4 (12q13-15 amplification) are co-amplified in well differentiated liposarcoma. The response to current therapies is poor. As in (Table 2) several patients with MDM2 aberration benefitted clinically from MDM2 inhibitors in early phase clinical trials. Clinical trials are underway in liposarcoma using MDM2 inhibitors either singly or in combination with CDK4 inhibitors. The efficacy of these agents as a group are to be determined soon.

Our study confirms several previously described overexpressed pathways in sarcomas such as MDM2-CKD4 and FRS2-FGF. Importantly it demonstrates that these are not unique to the previously described sarcomas, and indeed are present in other subtypes. This underscores the importance of NGS in all sarcoma patients to find these potentially actionable mutations. Additionally, it highlights the need for basket trials in sarcoma that are targeted to mutations and pathways rather than histologic subtypes. With properly designed trials, these could even be accepted for drug registration or expanded indications.

Limitations abound in a retrospective observational study such as ours. While we consider our census size to be adequate, there was a wide variety of subtypes. Many of these subtypes included a single individual making any kind of conclusion impossible. This is an unfortunate consequence of sarcoma heterogeneity. However, this created a distinct advantage in showing that certain pathways are deranged in diverse subtypes. Our definition of an actionable mutation is based on aggregation of myriad studies. The true clinical utility of any given drug to target a particular mutation is not known until a prospective trial is done. However, our observational study was able to demonstrate at least anecdotal evidence of clinical benefit from targeted therapy.

In conclusion, based on our findings we believe that future studies in sarcomas should be guided by NGS and actionable alterations rather than histologic subtypes. Sarcomas are lacking in development of targeted therapy, but we demonstrate that there are myriad targets with novel therapeutic potential. We believe that personalization will shape future therapy in oncology. A rare and heterogeneous neoplasm like sarcoma would especially benefit from such a personalized approach.

## MATERIALS AND METHODS

The electronic medical records of 102 diverse sarcoma patients were reviewed and history, laboratory and clinical findings were abstracted. These patients were referred to the Investigational Therapeutics Department at MD Anderson Cancer Center (MDACC). All pathology had previously been reviewed and confirmed by an MDACC pathologist with experience in bone and soft-tissue sarcomas. Therapies differed based on clinical trial opportunities at date of visit. All patients had a commercially available comprehensive genomic panel from Foundation Medicine (FoundationOne, http://www.foundationone.com). Profiling could have been performed as part of prior care. Otherwise, genomic profiling was performed upon phase 1 clinic presentation.

Patient attributes noted from the chart included age, sex, race, tumor histology, and whether the biopsy was from primary tumor or metastasis. Additional data recorded include type of investigational therapy and start date, as well as best overall response and duration of response based on Response Evaluation Criteria in Solid Tumors (RECIST V1.1). Date of death or last follow-up were also noted.

Each of the represented clinical trials in this review were independently approved by the MD Anderson institutional review board (IRB) and patients provided written consent to be treated with the corresponding investigational therapy. This retrospective review was also approved by the MD Anderson IRB.

NGS was performed by Foundation Medicine (FoundationOne, http://www.foundationone.com), a clinical grade CLIA-approved NGS test analyzing 236 or 315 cancer-related genes in at least 50ng of DNA from routine formalin-fixed and paraffin-embedded (FFPE) clinical specimens [42].

Actionable gene alteration was defined as any gene alteration that is either directly targeted or a pathway component of a directly targeted gene by an approved or investigational drug [43] (Table 5).

**Table 5:** FDA-approved drugs that target genes with published evidence

Gene	Drugs
ABL1	Bosutinib, Dasatinib, Imatinib, Sorafenib, Vandetanib
ALK	Alectinib, Ceritinib, Crizotinib
AR	Bicalutamide, Enzalutamide, Flutamide
BRAF	Dabrafenib, Regorafenib, Sorafenib
CDK4	Palbociclib
CSF1R	Sunitinib
DDR2	Dasatinib
DNMT3A	Azacitidine
EGFR	Afatinib, Cetuximab, Erlotinib, Gefitinib, Lapatinib, Osimertinib, Panitumumab, Vandetanib
FGFR1	Lenvatinib, Pazopanib, Regorafenib, Sorafenib, Sunitinib
FGFR2	Lenvatinib, Pazopanib, Regorafenib, Sorafenib, Sunitinib
FLT4	Axitinib, Cabozantinib, Lenvatinib, Pazopanib, Sorafenib, Sunitinib, Vandatenib
JAK1	Ruxolitinib
JAK2	Ruxolitinib
JAK3	Ruxolitinib, Tofacitinib
KDR	Axitinib, Cabozantinib, Lenvatinib, Pazopanib, Ramucirumab, Regorafenib, Sorafenib, Sunitinib, Vandetanib
KIT	Axitinib, Cabozantinib, Dasatinib, Imatinib, Lenvatinib, Pazopanib, Regorafenib, Sorafenib, Sunitinib
MAP2K2	Trametinib
MET	Cabozantinib, Crizotinib
MPL	Eltrombopag Olamine, Romiplostim
MTOR	Everolimus, Sirolimus, Temsirolimus
NTRK1	Crizotinib, Regorafenib
PDGFRA	Axitinib, Dasatinib, Imatinib, Lenvatinib, Pazopanib, Regorafenib, Sorafenib, Sunitinib
PDGFRB	Axitinib, Cabozantinib, Dasatinib, Imatinib, Lenvatinib, Pazopanib, Regorafenib, Sorafenib, Sunitinib
ROS1	Ceritinib, Crizotinib

Electronic medical records were reviewed for above mentioned demographic and diagnostic data. The respective molecular diagnostic reports were reviewed for alterations with a potentially actionable mutation either on-label, off-label, or in clinical trials. If patients received treatment with an investigational therapeutic, this was recorded along with the response.

## Abbreviations

PD: progressive disease
PR: partial response
SD: stable disease
CR: complete response
ER: estrogen receptor
IHC: immunohistochemistry
Dx: diagnosed
WT: wild type
FM: foundation medicine
